# A new estimator of between study variance of standardized mean difference in meta-analysis

**DOI:** 10.1371/journal.pone.0308628

**Published:** 2024-11-01

**Authors:** Ramlah H. Albayyat, Hajar S. Aljohani, Dalia K. Alnagar

**Affiliations:** 1 Department of Mathematics, Northern Border University, Arar, Saudi Arabia; 2 Department of Statistics, University of Tabuk, Tabuk, Saudi Arabia; ICAR Indian Agricultural Statistics Research Institute, INDIA

## Abstract

Meta-analysis is a statistical technique that combines the results of different environmental experiments regarding the populations, location, time, and so on. These results will differ more than the within-study variance, and the true effects being evaluated differ between studies. Thus, heterogeneity is present and should be measured. There are different estimators that were introduced to estimate between-study variance, which has received a lot of criticism from previous researchers. All of the estimators encountered the same problem, which was the correlation. To minimize the potential biases caused by interventions between the three estimators (i.e., overall effect size, within-study variance, and between-study variance), we proposed a new measure of heterogeneity known as the Environmental Effect Ratio (EER), the treatment-by-lab variability relative to the experimental error, under individual participant data (IPD) using the linear mixed model approach. We assume different between-study variances instead of constant between-study variances. The simulation of this study focuses on the performance of meta-analyses with small sample sizes. We compared our proposed estimator under two different expressions (${\hat{\tau }}_{1EER}^{2}$, and ${\hat{\tau }}_{2EER}^{2}$) with the best estimator nominated from previous studies to determine which one is the best performance. Based on the findings, our estimator (${\hat{\tau }}_{2EER}^{2}$) was better for estimating between-study variance.

## 1 Introduction

Different experimental environment variations regarding the populations, location, time and so on contribute to a unique research environment in each experiment, making it difficult for another researcher to redo a study in the same way, even if both studies are conducted with high accuracy [1–3]. Meta-analysis is considered as the organization of the scientific chaos [4]. Researchers use meta-analysis, a statistical technique combining several independent studies’ findings, to tackle this issue [5, 6], as follows, first, converting the results into a common metric using an effect-size index known as the standardized mean difference (SMD) is necessary [7]. Second, a random-effects meta-analysis is usually considered, in which both within-study and between-study heterogeneity are estimated [8, 9]. Thus, the accurate estimation of within-study and between-study variances is crucial, as inaccurate estimation can compromise the validity of the synthesized SMD (calculated using the inverse variance weighting method) [7, 10–12]. Numerous methods have been put forward to measure the amount of between-study variance that varies in popularity and complexity; an S1 Table containing a list of these estimators and their abbreviations is provided. Estimating the between-study variance accurately can be quite challenging, especially when the number of studies and the number of units within a study are small. [9, 13–16]. Conflicting results were presented based on between-study variances, with values ranging from 0 to 24.56 (as shown in S2 Table) [17], despite some of these methods having similar methodologies. The majority of these methods are based on the method of moments [18]. The conflicting results may have been caused by either using aggregate data or assuming the heterogeneity is equal across all studies.

To address the concern mentioned above, a new estimator with two different expressions will be proposed by utilizing individual participant data (IPD); the critical assumption of heterogeneity is changed by assuming different between-study variances instead of identical. Our study starts by assuming that treatment effects vary randomly across labs. We will use the mixed model approach to propose the measure of heterogeneity known as the Environmental Effect Ratio (EER), the treatment-by-lab variability relative to the experimental error. The properties of this estimator minimize the potential biases caused by interventions between the three estimators (i.e., overall effect size, within-study variance and between-study variance).

We will compare our proposed estimator with the most effective estimator suggested by the previous researchers, which is the restricted maximum likelihood (REML) [19]. The study compares heterogeneity estimators based on MSE, Bias, and Proportion of zero.

This study is structured as follows: Section 2 introduced combining study-level effect size estimates and estimate variance components. In section 3, we discuss our techniques and derive a new estimator. Section 4 carried out a simulation study. Finally, we report our conclusions in section 5.

## 2 Methods

### 2.1 Combining study-level effect size estimates

There are two models involved in meta-analysis, the fixed-effect model, and the random-effects model. The fixed-effect model makes the assumption that all studies under consideration share a single true effect size *δ*, whereas the random-effects model considers the possibility of variation in the true effect size among studies due to their differences (heterogeneity) *δ*_*j*_. [20]. This study focuses on the random-effects model.

The study-level effect sizes *δ*_*j*_ are commonly estimated by Cohen’s *d*, or Hedges’ *g* [21]. However, because the estimation by Cohen’s *d* (${\hat{\delta }}_{j}$) is biased in studies with small sample sizes, Hedges’ *g* proposed a small-sample bias-corrected estimator $E\left({\hat{\delta }}_{j}\right)=\delta_{j}/J_{j}$ [22]. The Hedges’ *g* estimator is given as follows.

$$\begin{array}{c} {\hat{\delta }}_{j}=J_{j}(\frac{{\bar{Y}}_{1j\cdot }-{\bar{Y}}_{2j\cdot }}{S_{p.j}}) \end{array}$$ (1)

where ${\bar{Y}}_{1j\cdot }$, and ${\bar{Y}}_{2j\cdot }$ define as the mean of first group, and the second group respectively. The *S*_*p*.*j*_ is the pool of the standard deviation, and *J*_*j*_ is given by

$$\begin{array}{c} J_{j}=\frac{\Gamma (\left(n_{1j}+n_{2j}-2\right)/2)}{\sqrt{(n_{1j}+n_{2j}-2)/2}\;\Gamma \left[\left(\left(n_{1j}+n_{2j}-2\right)-1\right)/2\right]} \end{array}$$ (2)

and *S*_*p*.*j*_ pooled standard deviation for the effect size in study *j*:

$$\begin{array}{c} S_{p.j}=\sqrt{\frac{\left(n_{1j}-1\right)S_{1j}^{2}+\left(n_{2j}-1\right)S_{2j}^{2}}{n_{1j}+n_{2j}-2}}. \end{array}$$ (3)

The exact distribution of $\sqrt{n_{1j}n_{2j}/\left(n_{1j}+n_{2j}\right)}\;{\hat{\delta }}_{j}$ is a non-central t-distribution with *n*_1*j*_ + *n*_2*j*_ − 2 degrees of freedom, and non-centrality parameter $\sqrt{n_{1j}n_{2j}/\left(n_{1j}+n_{2j}\right)}\;\delta_{j}$. In practice, however, it is often approximated using a normal distribution if the number of units in study *j* is sufficiently large

$$\begin{array}{c} {\hat{\delta }}_{j}∼N\left(\delta_{j},\sigma_{j}^{2}\right),\;\;\;\;j=1,\ldots ,m, \end{array}$$ (4)

$$\begin{array}{c} \delta_{j}∼N\left(\Delta ,\tau^{2}\right). \end{array}$$ (5)

where $\sigma_{j}^{2}$ indicates within study variance, and *τ*^2^ between study variance respectively.

The study-level effect size is commonly combined using a weighted average effect size, expressed as the following.

$$\begin{array}{c} \hat{\Delta }=\frac{\sum_{j=1}^{m}{\hat{w}}_{j}\;{\hat{\delta }}_{j}}{\sum_{j=1}^{m}{\hat{w}}_{j}}. \end{array}$$ (6)

Under the random-effects meta-analysis model, the weights are given by ${\hat{w}}_{j}=1/\left(s_{j}^{2}+{\hat{\tau }}^{2}\right)$, where $s_{j}^{2}$ is an estimate of the within-study variance $\sigma_{j}^{2}$ and ${\hat{\tau }}^{2}$ is an estimate of the between-study variance.

### 2.2 Estimating variance components

Using properties of the non-central *t*-distribution, the exact variance of the within-study variance of ${\hat{\delta }}_{j}$ conditional on the study-level effect size *δ*_*j*_ is

$$\begin{array}{c} \sigma_{j}^{2}=Var\left({\hat{\delta }}_{j}|\delta_{j}\right)=\frac{n_{1j}+n_{2j}-2}{n_{1j}+n_{2j}-4}(J_{j}^{2}\;\tilde{n_{j}}+\frac{\gamma_{j}\;J_{j}^{2}\;\delta_{j}^{2}}{\left(n_{1j}+n_{2j}-2\right)}) \end{array}$$ (7)

where

$$\begin{array}{c} \gamma_{j}=\left(n_{1j}+n_{2j}-2\right)-\frac{\left(n_{1j}+n_{2j}-4\right)}{J_{j}^{2}}. \end{array}$$ (8)

and $\tilde{n_{j}}=1/n_{1j}+1/n_{2j}$ and *J*^2^ is defined in Eq (2).

Estimates of this variance depend mainly on how these effect sizes are estimated and the sample sizes within each study. A natural estimator for $\sigma_{j}^{2}$ can be obtained simply by replacing *δ*_*j*_ with an estimate of this quantity.

Although Hedges’ g’s estimator is (nearly) unbiased within each individual study, the synthesized Hedges’ g gives biased results due to a correlation between the effect sizes ${\hat{\delta }}_{j}^{2}$ and their within-study variances $s_{j}^{2}$. Therefore, studies with little replication require a method that weights each study by the inverse of the mean-adjusted error variance to eliminate or substantially reduce the bias [23], as the following

$$\begin{array}{c} s_{j}^{2}=J_{j}^{2}(\tilde{n_{j}}+\frac{{\left(\sum_{j=1}^{m}{\hat{\delta }}_{j}/m\right)}^{2}}{2(n_{1j}+n_{2j})}) \end{array}$$ (9)

In addition, after considering all potential adjustments to within-study variance (as shown in S4 Table), the Eq 9 was also recommended to eliminate the bias [24].

In terms of between-study variance, S3 Table shows that most researchers recommended using the restricted maximum likelihood (REML) [19] because they perform better than others [25–27].

$$\begin{array}{c} {\hat{\tau }}_{REML}^{2}=\frac{\sum_{j=1}^{m}{\hat{w_{j}}}^{2}({\left({\hat{\delta }}_{j}-{\hat{\Delta }}_{REML}\right)}^{2}\;-s_{j}^{2})}{\sum_{j=1}^{m}{\hat{w_{j}}}^{2}}+\frac{1}{\sum_{j=1}^{m}\hat{w_{j}}} \end{array}$$ (10)

where

$$\begin{array}{c} \hat{w_{j}}=\frac{1}{{\hat{\tau }}_{REML}^{2}+s_{j}^{2}} \end{array}$$ (11)

$$\begin{array}{c} {\hat{\Delta }}_{REML}=\frac{\sum_{j=1}^{m}{\hat{w}}_{j}\;{\hat{\delta }}_{j}}{\sum_{j=1}^{m}{\hat{w}}_{j}}. \end{array}$$ (12)

## 3 New estimator of between-study variance using linear mixed model

Several different estimators were proposed to estimate between-study variance, which has received a lot of criticism from previous researchers [14, 28]. All estimators faced the same issue, which was the correlation. To minimize the potential biases caused by interventions between the three estimators (i.e., overall effect size, within-study variance, and between-study variance), a new estimator will be derived using individual participants’ data. Previously, we discussed how meta-analysis techniques estimate variance components: aggregate data are used to estimate between-studies, while observations are used to estimate within-study variance. However, in this study, to analyze multi-lab data efficiency, the observations will be used to estimate the variance components by deriving candidate measures of heterogeneity known as the Environmental Effect Ratio (EER) [2]. The EER is the ratio of the standard deviations of the environment treatment interaction and error. We will consider the special case in which the EER from each study is known and equal.

### 3.1 The proposed estimator $\tau_{EER.j}^{2}$

In a previous study [29], both component variances were estimated assuming that the observations on the jth experiment from the experimental and control groups, respectively, were fixed for j to estimate within-study variance and, after that, used to estimate between-study variance. In this study, we consider the jth experiments are randomly since each of the m studies is conducted within its own lab.

The heterogeneity measure, EER, is derived using the mixed linear model approach. The jth experiments are considered random because each of the m studies is conducted within its own lab. We begin with the following model of response for multi-lab studies:

$$\begin{array}{c} Y_{ijk}=\mu_{i}+\theta_{j}+\zeta_{ij}+\varepsilon_{ijk},\;\;\;\;i=1,2,\;\;\;\;j=1,\ldots ,m,\;\;\;\;k=1,..,n_{ij}. \end{array}$$ (13)

Here, *Y*_*ijk*_ is the response for the *k*^th^ observation given treatment *i* in study *j*; *μ*_*i*_ is the population mean for treatment *i*; *θ*_*j*_ is a lab effect that impacts all responses in study *j*; *ζ*_*ij*_ is a lab-by-treatment effect that impacts responses given treatment *i* in study *j*; and *ε*_*ijk*_ is the experimental error. The *θ*_*j*_, *ζ*_*ij*_ and *ε*_*ijk*_ random variables are assumed to be independent, where

$$\begin{array}{c} \theta_{j}∼N\left(0,\sigma_{\theta }^{2}\right),\;\;\;\;\;\;\zeta_{ij}∼N\left(0,\sigma_{\zeta }^{2}\right), \end{array}$$ (14)

where $\sigma_{\theta }^{2}$ is the variance of the lab effect, and $\sigma_{\zeta }^{2}$ is the variance of a lab-by-treatment effect.

Under these assumptions Eq (14), the true *study-level effect size* for lab *j* is

$$\begin{array}{c} \delta_{j}∼N(\Delta ,2\;{\left(\frac{\sigma_{\zeta }}{\sigma_{e}}\right)}^{2}). \end{array}$$ (15)

In the replicability literature, the *σ*_*ζ*_/*σ*_*e*_ term may be referred to as the *environmental effect ratio* (EER) [2].

This study accounts for differing environments across labs when estimating effect sizes (*θ*_*j*_ ≠ 0), thus, we will have the following models:

$$\begin{array}{c} {\bar{Y}}_{1j\cdot }=\mu_{1}+\theta_{j}+\zeta_{1j}+{\bar{\varepsilon }}_{1j\cdot } \end{array}$$ (16)

$$\begin{array}{c} {\bar{Y}}_{2j\cdot }=\mu_{2}+\theta_{j}+\zeta_{2j}+{\bar{\varepsilon }}_{2j\cdot } \end{array}$$ (17)

where ${\bar{Y}}_{1j\cdot }$ and ${\bar{Y}}_{2j\cdot }$ are independent and identically distributed. The mathematical feature investigates the mean and variance of ${\hat{\delta }}_{j}$. Since, in reality, the ${\hat{\delta }}_{j}$ is a non-central t-distributed random variable, we need to reformulate the ${\hat{\delta }}_{j}$ as the following

$$\begin{array}{c} T=\frac{Z+\mu }{\sqrt{V/\nu }} \end{array}$$ (18)

where T is a non-central t distribution, Z and V are a normal and a Chi-squared distribution, respectively, *ν* is the degrees of freedom, and *μ* is a non-centrality parameter.

To find Z, using Eqs (16) and (17)

$$\begin{array}{c} {\bar{Y}}_{1j\cdot }-{\bar{Y}}_{2j\cdot }∼N\left(\mu_{1}-\mu_{2},\;2\;\sigma_{\zeta }^{2}+\;{\tilde{n}}_{j}\;\sigma_{e}^{2}\right) \end{array}$$

where

$$\begin{array}{c} {\tilde{n}}_{j}=\frac{1}{n_{1j}}+\frac{1}{n_{2j}} \end{array}$$

$$\begin{array}{c} Z=\frac{\left({\bar{Y}}_{1j\cdot }-{\bar{Y}}_{2j\cdot }\right)-\left(\mu_{1}-\mu_{2}\right)}{\sqrt{2\;\sigma_{\zeta }^{2}+\;{\tilde{n}}_{j}\sigma_{e}^{2}}}∼N\left(0,1\right) \end{array}$$ (19)

The V can be obtained by

$$\begin{array}{c} V=\frac{\left(n_{1j}-1\right)s_{1j}^{2}+\left(n_{2j}-1\right)s_{2j}^{2}}{\sigma_{e}^{2}\left(n_{1j}+n_{2j}-2\right)}∼\frac{1}{n_{1j}+n_{2j}-2}\chi_{n_{1j}+n_{2j}-2}^{2} \end{array}$$ (20)

Modifying ${\hat{\delta }}_{j}$ to be in the similar form as T

$$\begin{array}{c} {\hat{\delta }}_{j}=\frac{{\bar{Y}}_{1j\cdot }-{\bar{Y}}_{2j\cdot }}{S_{p.j}}=\frac{(\left({\bar{Y}}_{1j\cdot }-{\bar{Y}}_{2j\cdot }\right)-\left(\mu_{1}-\mu_{2}\right)+\left(\mu_{1}-\mu_{2}\right))/\sqrt{2\;\sigma_{\zeta }^{2}+\;{\tilde{n}}_{j}\sigma_{e}^{2}}}{S_{p.j}/\sqrt{2\;\sigma_{\zeta }^{2}+\;{\tilde{n}}_{j}\sigma_{e}^{2}}} \end{array}$$

After simplification by using Eqs (19) and (20), we have

$$\begin{array}{c} {\hat{\delta }}_{j}=\sqrt{2\;\left(\sigma_{\zeta }^{2}/\sigma_{e}^{2}\right)+\;{\tilde{n}}_{j}}\;\;(\frac{Z+\mu }{\sqrt{V/df}}) \end{array}$$ (21)

where

$$\begin{array}{c} \mu =\Delta /\sqrt{2\;\left(\sigma_{\zeta }^{2}/\sigma_{e}^{2}\right)+\;{\tilde{n}}_{j}} \end{array}$$

By using the moment properties of the non-central t distribution, the exact mean and component variances are

$$\begin{array}{c} E\left({\hat{\delta }}_{j}\right)=\frac{\Delta }{J_{j}} \end{array}$$ (22)

$$\begin{array}{c} var\left({\hat{\delta }}_{j}\right)=[\frac{2\;\left(\sigma_{\zeta }^{2}/\sigma_{e}^{2}\right)\left(n_{1j}+n_{2j}-2\right)}{\left(n_{1j}+n_{2j}-4\right)}]+[\frac{\left(n_{1j}+n_{2j}-2\right)\;\left({\tilde{n}}_{j}+\Delta^{2}\right)}{\left(n_{1j}+n_{2j}-4\right)}-\frac{\Delta^{2}}{J_{j}^{2}}] \end{array}$$ (23)

$$\begin{array}{c} var\left({\hat{\delta }}_{j}\right)=\tau_{EER.j}^{2}+\sigma_{j}^{2} \end{array}$$

where the quantity $\sigma_{\zeta }^{2}/\sigma_{e}^{2}$ is *EER*^2^. Therefore, the critical assumption of *τ*^2^ should be changed by assuming there are between-study variances ($\tau_{EER.j}^{2}$) instead of between-study variance (*τ*^2^), and $\sigma_{j}^{2}$ is the within study variance, as the previous study.

Next, multiplying Eq (23) by $J_{j}^{2}$ since $\;J_{j}{\hat{\delta }}_{j}$ is unbiased for *δ*_*j*_, we have

$$\begin{array}{c} \tau_{EER.j}^{2}=\frac{2\;J_{j}^{2}\left(n_{1j}+n_{2j}-2\right)}{\left(n_{1j}+n_{2j}-4\right)}\;EER^{2} \end{array}$$ (24)

Since the within-study variance was used without considering the term (*n*_1*j*_ + *n*_2*j*_ − 2)/(*n*_1*j*_ + *n*_2*j*_ − 4), see Eq (9), this consideration will be taken into account when estimating between-study variance by suggestion two expressions $\tau_{1EER.j}^{2}$ which is as given in Eq (24) and $\tau_{2EER.j}^{2}$ as the following

$$\begin{array}{c} \tau_{2EER.j}^{2}=2\;J_{j}^{2}\;EER^{2} \end{array}$$ (25)

Obviously, $\tau_{1EER.j}^{2}$ and $\tau_{2EER.j}^{2}$ are independent on both effect size and between study variance as the previous study.

### 3.2 Unbiased estimates of $\tau_{rEER.j}^{2}$

The mean, variance, and an unbiased estimate of $\tau_{rEER.j}^{2},\;\;r=1,2$ are obtained using the F distribution. The ratio of the environment treatment to the error experiment for randomized complete block experiments with m replicates can be expressed as [30]

$$F=\frac{1+m{\hat{EER}}^{2}}{1+mEER^{2}}$$

(i) The $\tau_{1EER.j}^{2}$ can be expressed as

$$\begin{array}{c} {\hat{EER}}^{2}={EER}^{2}\frac{df_{2}}{df_{2}-2}+\frac{2}{m(df_{2}-2)} \end{array}$$

Therefore,

$$\begin{array}{c} {\hat{\tau }}_{1EER.j}^{2}=\tau_{1EER.j}^{2}\frac{df_{2}}{df_{2}-2}+\frac{4\;J_{j}^{2}\left(n_{1j}+n_{2j}-2\right)}{m\left(df_{2}-2\right)\left(n_{1j}+n_{2j}-4\right)} \end{array}$$

where *df*_2_ is the degrees of freedom for error variance. Therefore, an unbiased estimate of $\tau_{1EER.j}^{2}$ is

$$\begin{array}{c} \tau_{1EER.j}^{2}={\hat{\tau }}_{1EER.j}^{2}\frac{df_{2}-2}{df_{2}}-\frac{4\;J_{j}^{2}\left(n_{1j}+n_{2j}-2\right)}{m\;df_{2}\;\left(n_{1j}+n_{2j}-4\right)} \end{array}$$

(ii) The $\tau_{2EER.j}^{2}$ can be expressed as

$$\begin{array}{c} {\hat{\tau }}_{2EER.j}^{2}=\tau_{2EER.j}^{2}\frac{df_{2}}{df_{2}-2}+\frac{4\;J_{j}^{2}}{m(df_{2}-2)} \end{array}$$

where *df*_2_ is the degrees of freedom for error variance. Therefore, an unbiased estimate of $\tau_{2EER.j}^{2}$ is

$$\begin{array}{c} \tau_{2EER.j}^{2}={\hat{\tau }}_{2EER.j}^{2}\frac{df_{2}-2}{df_{2}}-\frac{4\;J_{j}^{2}}{m\;df_{2}\;} \end{array}$$

### 3.3 Between-study variance ${\hat{\tau }}_{rEER}^{2}$ under multi-lab studies

To analyze multi-lab data efficiency, the observations will be used to estimate between-study variance. The expected value of the variance was used as reported by [31] as follows:

$$\begin{array}{c} {\hat{\tau }}_{rEER}^{2}=\frac{1}{m}\sum_{j=1}^{m}\;{\hat{\tau }}_{rEER.j}^{2}\;\;\;\;r=1,2 \end{array}$$

The proposed estimator is unaffected by neither the within-study variance nor effect size. It minimizes the potential biases and Mean Square Error (MSE) caused by interventions, as shown in the section 4.

## 4 Simulation and result

In this section, we will use individual participant data (IPD) to evaluate the performances of estimators $\tau_{REML}^{2}$ $\tau_{1EER}^{2}$, and $\tau_{2EER}^{2}$, to determine the best estimator based on Bias, MSE, and proportion of zero. The proposed estimates will be obtained using the *lme* function, the nlme package [32], and lme4 package [33], with REML method. However, for estimating $\tau_{REML}^{2}$, own code will be utilized. The simulations for all estimators are carried out in R.

The study used a simulation with 5000 repetitions for each combination under different scenarios of *n*_*ij*_, Δ, *τ*^2^, and m. Multi-lab data ranged between five and thirty studies. Each study compares a treatment group with a control group. We select each sample sizes *n*_*ij*_ randomly,(5–15,10-20,50–70), and generate observations for each group using *μ*_*i*_ + *ζ*_*ij*_ + *θ*_*j*_ + *e*_*ijk*_. The data of *ζ*_*ij*_, *θ*_*j*_ and *e*_*ijk*_ are generated as independent and identically distributed with $\zeta_{ij}∼N\left(0,\sigma_{\zeta }^{2}\right)$, $\theta_{j}∼N\left(0,\sigma_{\theta }^{2}\right)$, and $e_{ijk}∼N\left(0,\sigma_{e}^{2}\right)$, respectively, *i* = 1, 2 and *j* denotes the number of studies. The value of $\sigma_{e}^{2}$ is set to one. The categorized values of Δ are defined as zero and 0.5. The *τ*^2^ values are selected as 0.05, 0.3, and 1.

**Fig 1:**
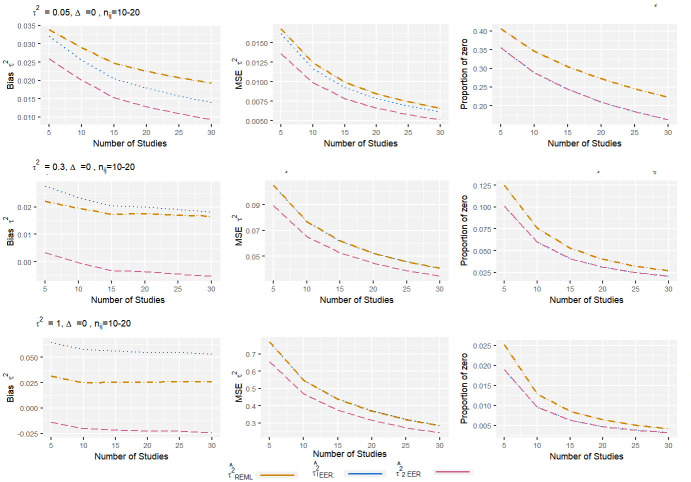
Bias, MSE, and proportion of zero of heterogeneity variance estimates in standardized mean difference with small sample size and no effect size. The true between-study variance increased from 0.05 to 1.

**Fig 2:**
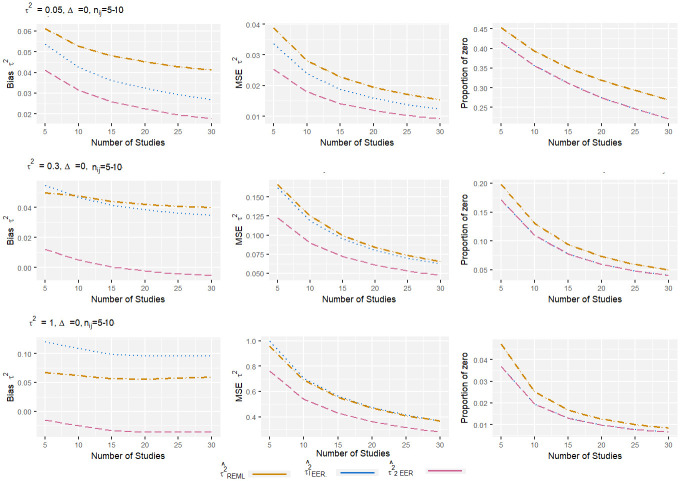
Bias, MSE, and proportion of zero of heterogeneity variance estimates in standardized mean difference with very small sample size and no effect size. The true between-study variance increased from 0.05 to 1.

**Fig 3:**
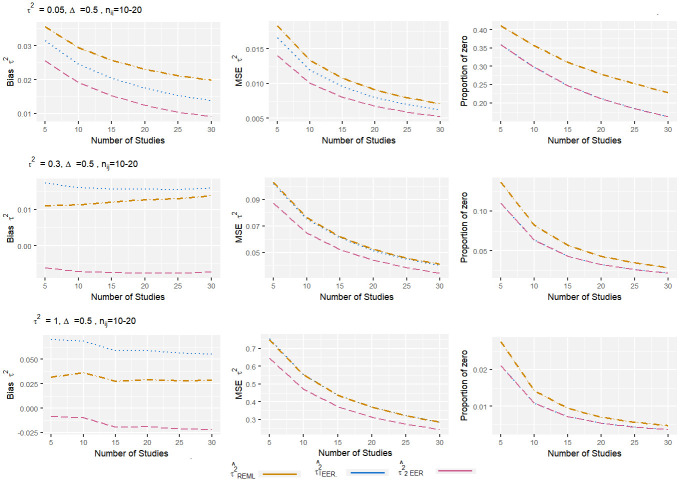
Bias, MSE, and proportion of zero of heterogeneity variance estimates in standardized mean difference with small sample size and medium effect size. The true between-study variance increased from 0.05 to 1.

**Fig 4:**
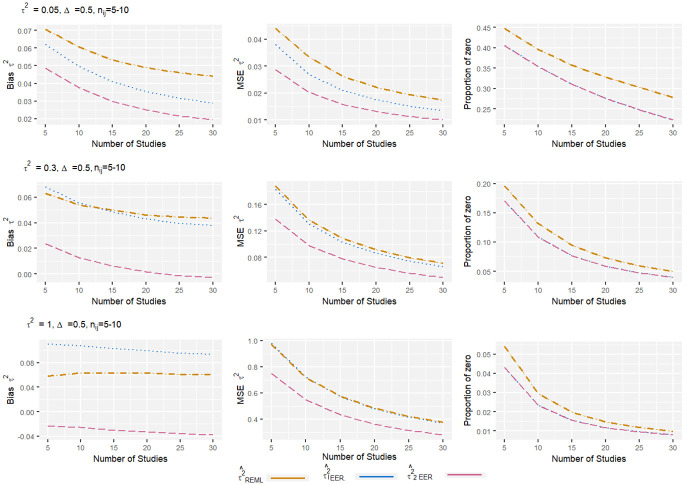
Bias, MSE, and proportion of zero of heterogeneity variance estimates in standardized mean difference with very small sample size and medium effect size. The true between-study variance increased from 0.05 to 1.

From the Figs 1–4, it can be observed that, for the low level of heterogeneity (*τ*^2^ = 0.05), the $\tau_{1EER}^{2}$ and ${\hat{\tau }}_{2EER}^{2}$ estimators produced slightly lower MSE compared to the traditional estimator ($\tau_{REML}^{2}$). The negligible difference in MSE among these estimators was due to their high proportion of zeros. When *τ*^2^ increased to 1, the proportion of zero decreased significantly, and the ${\hat{\tau }}_{2EER}^{2}$ estimator produced clearly less MSE compared to others. Regarding bias, the estimator $\tau_{2EER}^{2}$ consistently produced the lowest amount of bias, while the $\tau_{1EER}^{2}$ estimator performed worst in some scenarios. When the number of studies increased, the properties of all the estimators improved. The finding shows that the estimator ${\hat{\tau }}_{2EER}^{2}$ consistently yields the lowest MSE, bias, and proportion of zero for all combinations of parameters.

## 5 Conclusion

The presence of heterogeneity in a meta-analysis indicates that the true effects vary between studies, which is a crucial part of meta-analysis. This study introduced a new estimator known as the Environmental Effect Ratio (EER) with two expressions, $\tau_{1EER}^{2}$ and $\tau_{2EER}^{2}$, using the mixed model technique to estimate the between-study variance of standardized mean differences from individual participant data. Our approach has revealed that each study has different variance components, contradicting previous research that assumed sets of studies to have identical heterogeneity. We compared our proposed estimator with the best estimator nominated from previous studies (The traditional REML method) to determine which is more efficient for analyzing the multi-lab study under meta-analysis. The evaluations are based on MSE, Bias, and Proportion of zero. Simulation results showed that the estimator ${\hat{\tau }}_{2EER}^{2}$ consistently yields the lowest MSE, bias, and proportion of zero for all combinations of parameters. To use this estimator for meta-analysis, it’s important for the research findings to include the amount of EER; this will ensure the accuracy of the estimator and help researchers make informed decisions.

## Supporting information

S1 Table Abbreviations of the estimators for the between-study variance. (PDF)

S2 Table Conflicting results of estimating the heterogeneity variance. (PDF)

S3 Table Comparison of between-study variance variance estimators. (PDF)

S4 Table Estimators of within-study variance. (PDF)

## References

[1] Kontopantelis E, Springate DA, Reeves D. A re-analysis of the Cochrane Library data: the dangers of unobserved heterogeneity in meta-analyses. PloS one. 2013;8(7):e69930. doi: 10.1371/journal.pone.0069930 23922860 PMC3724681

[2] Higgins JJ, Higgins MJ, Lin J. From one environment to many: The problem of replicability of statistical inferences. The American Statistician. 2021;75(3):334–342. doi: 10.1080/00031305.2020.1829047

[3] Loken E, Gelman A. Measurement error and the replication crisis. Science. 2017;355(6325):584–585. doi: 10.1126/science.aal3618 28183939

[4] Hunt M. How science takes stock: The story of meta-analysis. Russell Sage Foundation; 1997.

[5] Haidich AB. Meta-analysis in medical research. Hippokratia. 2010;14(Suppl 1):29. 21487488 PMC3049418

[6] Sharpe D, Poets S. Meta-analysis as a response to the replication crisis. Canadian Psychology/Psychologie canadienne. 2020;61(4):377. doi: 10.1037/cap0000215

[7] Sánchez-Meca J, Marin-Martinez F. Weighting by inverse variance or by sample size in meta-analysis: A simulation study. Educational and Psychological Measurement. 1998;58(2):211–220. doi: 10.1177/0013164498058002005

[8] DerSimonian R, Laird N. Meta-analysis in clinical trials. Controlled clinical trials. 1986;7(3):177–188. doi: 10.1016/0197-2456(86)90046-2 3802833

[9] Higgins JP, Thompson SG, Spiegelhalter DJ. A re-evaluation of random-effects meta-analysis. Journal of the Royal Statistical Society: Series A (Statistics in Society). 2009;172(1):137–159. doi: 10.1111/j.1467-985X.2008.00552.x 19381330 PMC2667312

[10] Sidik K, Jonkman JN. Robust variance estimation for random effects meta-analysis. Computational Statistics & Data Analysis. 2006;50(12):3681–3701. doi: 10.1016/j.csda.2005.07.019

[11] Hamman EA, Pappalardo P, Bence JR, Peacor SD, Osenberg CW. Bias in meta-analyses using Hedges’d. Ecosphere. 2018;9(9):e02419. doi: 10.1002/ecs2.2419

[12] Marin-Martinez F, Sánchez-Meca J. Weighting by inverse variance or by sample size in random-effects meta-analysis. Educational and Psychological Measurement. 2010;70(1):56–73. doi: 10.1177/0013164409344534

[13] Turner RM, Davey J, Clarke MJ, Thompson SG, Higgins JP. Predicting the extent of heterogeneity in meta-analysis, using empirical data from the Cochrane Database of Systematic Reviews. International journal of epidemiology. 2012;41(3):818–827. doi: 10.1093/ije/dys041 22461129 PMC3396310

[14] Novianti PW, Roes KC, van der Tweel I. Estimation of between-trial variance in sequential meta-analyses: a simulation study. Contemporary clinical trials. 2014;37(1):129–138. doi: 10.1016/j.cct.2013.11.012 24321246

[15] IntHout J, Ioannidis JP, Borm GF, Goeman JJ. Small studies are more heterogeneous than large ones: a meta-meta-analysis. Journal of clinical epidemiology. 2015;68(8):860–869. doi: 10.1016/j.jclinepi.2015.03.017 25959635

[16] Chung Y, Rabe-Hesketh S, Choi IH. Avoiding zero between-study variance estimates in random-effects meta-analysis. Statistics in medicine. 2013;32(23):4071–4089. doi: 10.1002/sim.5821 23670939

[17] Guo R, Pittler MH, Ernst E. Hawthorn extract for treating chronic heart failure. Cochrane Database of Systematic Reviews. 2008;37(1). 18254076 10.1002/14651858.CD005312.pub2 PMC11753770

[18] van Aert RC, Jackson D. Multistep estimators of the between-study variance: The relationship with the Paule-Mandel estimator. Statistics in medicine. 2018;37(17):2616–2629. doi: 10.1002/sim.7665 29700839 PMC6055723

[19] Viechtbauer W. Bias and efficiency of meta-analytic variance estimators in the random-effects model. Journal of Educational and Behavioral Statistics. 2005;30(3):261–293. doi: 10.3102/10769986030003261

[20] Dettori JR, Norvell DC, Chapman JR. Fixed-effect vs random-effects models for meta-analysis: 3 points to consider. Global Spine Journal. 2022;12(7):1624–1626. doi: 10.1177/21925682221110527 35723546 PMC9393987

[21] Lin L, Aloe AM. Evaluation of various estimators for standardized mean difference in meta-analysis. Statistics in Medicine. 2021;40(2):403–426. doi: 10.1002/sim.8781 33180373 PMC7770064

[22] Hedges LV. Distribution theory for Glass’s estimator of effect size and related estimators. journal of Educational Statistics. 1981;6(2):107–128. doi: 10.3102/10769986006002107

[23] Doncaster CP, Spake R. Correction for bias in meta-analysis of little-replicated studies. Methods in Ecology and Evolution. 2018;9(3):634–644. doi: 10.1111/2041-210X.12927 29938012 PMC5993351

[24] Albayyat R. On the use of meta-analysis techniques for multi-lab experiments. Kansas State University; 2023.

[25] Panityakul T, Bumrungsup C, Knapp G. On Estimating Residual Heterogeneity in Random-Effects Meta-Regression: A Comparative Study. J Stat Theory Appl. 2013;12(3):253–265. doi: 10.2991/jsta.2013.12.3.4

[26] Langan D, Higgins JP, Jackson D, Bowden J, Veroniki AA, Kontopantelis E, et al. A comparison of heterogeneity variance estimators in simulated random-effects meta-analyses. Research synthesis methods. 2019;10(1):83–98. doi: 10.1002/jrsm.1316 30067315

[27] Hönekopp J, Linden AH. Heterogeneity estimates in a biased world. PloS one. 2022;17(2):e0262809. doi: 10.1371/journal.pone.0262809 35113897 PMC8812955

[28] Langan D. Estimating the Heterogeneity Variance in a Random-Effects Meta-Analysis. University of York; 2015.

[29] Hedges LV. A random effects model for effect sizes. Psychological Bulletin. 1983;93(2):388. doi: 10.1037/0033-2909.93.2.388

[30] Federer WT. Evaluation of variance components from a group of experiments with multiple classifications. Iowa State University; 1948.

[31] Searle SR. Linear models. vol. 65. John Wiley & Sons; 1997.

[32] Pinheiro J, Bates D, DebRoy S, Sarkar D, Heisterkamp S, Van Willigen B, et al. Package ‘nlme’. Linear and nonlinear mixed effects models, version. 2017;3(1):274.

[33] Bates D, Mächler M, Bolker B, Walker S. Fitting linear mixed-effects models using lme4. arXiv preprint arXiv:14065823. 2014;.

