# Supplementary material for: A new estimator of between study variance of standardized mean difference in meta-analysis
Source: PLoS One. 2024 Nov 1;19(11):e0308628. doi: 10.1371/journal.pone.0308628 (PMC11530055; doi:10.1371/journal.pone.0308628)
Supplement: S1 Table — (PDF) [file pone.0308628.s001.pdf]

S1 Table. Abbreviations of the Estimators for the Between-Study Variance

Estimator	Abbreviations
1. DerSimonian and Laird	DL
2. Positive DerSimonian and Laird	DL_p
3. Two-step DerSimonian and Laird	DL_2
4. Cochran's ANOVA	CA
5. Two-step Cochran's ANOVA	PM_{CA}
6. Paule and Mandel	PM
7. Hartung and Makambi	HM
8. Hunter and Schmidt	HS
9. Maximum likelihood	ML
10. Restricted maximum likelihood	REML
11. Approximate restricted maximum likelihood	AREML
12. Sidik-Jonkman (CA initial estimate)	SJ_{CA}
13. Bayes estimators Rukhin Bayes	RB
14. Positive Rukhin Bayes	RB_p
15. Bayes Modal	BM
16. Bootstrap DerSimonian-Laird	DL_B
17. Malzahn, Böhning and Holling	MBH
18. Rukhin (zero prior)	B0
19. Rukhin (simple)	BP
20. Rukhin (alternate)	SB
21. Full Bayes	FB
22. Approximate Bayes	AB
23. Sidik and Jonkman	SJ
