# Supplementary material for: A new estimator of between study variance of standardized mean difference in meta-analysis
Source: PLoS One. 2024 Nov 1;19(11):e0308628. doi: 10.1371/journal.pone.0308628 (PMC11530055; doi:10.1371/journal.pone.0308628)
Supplement: S2 Table — (PDF) [file pone.0308628.s002.pdf]

S2 Table. Conflicting Results of Estimating the Heterogeneity Variance

Estimator	Between study variance
DL	6.56
ML	1.27
DL_P	6.56
REML	9.31
CA	0
ARML	7.16
PM	5.88
SJ	13.93
SJ_{CA}	0.01
HM	11.14
BP	24.56
HS	0.80
