# Supplementary material for: A new estimator of between study variance of standardized mean difference in meta-analysis
Source: PLoS One. 2024 Nov 1;19(11):e0308628. doi: 10.1371/journal.pone.0308628 (PMC11530055; doi:10.1371/journal.pone.0308628)
Supplement: S3 Table — (PDF) [file pone.0308628.s003.pdf]

S3 Table. comparison of between-study variance variance estimators

Reference	Estimators of between-study variance	Recommended	properties
[6]	HE, DL, ML, REML, SJ, and MP	PM	small sample size and a small number of studies (m=10)
		HE	large number of studies (m=30)
		REML followed by PM	large sample size and a small number of studies (m=10)
		PM followed by REML	large sample size and large number of studies (m=30)
[4]	HS, HE, DL, ML, and REML	REML	
		DL	when within-study variances are known and when the number of studies are large
[2]	DL_2 PM DL, HE, REML, SJ, and SJ_{HE}	DL_2 and PM	with different numbers of studies (M=10, 15, 20, 30, and 50.)
[1]		DL	when within-study variances are known and when the number of studies are large
[7]	HE, MM, ML, REML, EB, MV, MV_{vc}	HE	the number of studies is large ($m > 30$)
		EB and MV_{vc}	when the heterogeneity variance is moderate to large
[3]	CA, DL, PM, PM_{DL}, PM_{CA}, HM, HS, SJ, SJ_{CA}, ML, EML, B0, BP , and MBH	REML	
[5]	RAML, PM, and ML	REML	

References

1. Kontopantelis E, Springate DA, Reeves D. A re-analysis of the Cochrane Library data: the dangers of unobserved heterogeneity in meta-analyses. PloS one. 2013;8(7):e69930.

2. Novianti PW, Roes KC, van der Tweel I. Estimation of between-trial variance in

sequential meta-analyses: a simulation study. Contemporary clinical trials. 2014;37(1):129–138.

3. Langan D. Estimating the Heterogeneity Variance in a Random-Effects Meta-Analysis. University of York; 2015.

4. Viechtbauer W. Bias and efficiency of meta-analytic variance estimators in the random-effects model. Journal of Educational and Behavioral Statistics. 2005;30(3):261–293.

5. Albayyat R. On the use of meta-analysis techniques for multi-lab experiments. Kansas State University; 2023.

6. Panityakul T, Bumrungsup C, Knapp G. On Estimating Residual Heterogeneity in Random-Effects Meta-Regression: A Comparative Study. J Stat Theory Appl. 2013;12(3):253–265.

7. Sidik K, Jonkman JN. A comparison of heterogeneity variance estimators in combining results of studies. Statistics in medicine. 2007;26(9):1964–1981.
