# Supplementary material for: A new estimator of between study variance of standardized mean difference in meta-analysis
Source: PLoS One. 2024 Nov 1;19(11):e0308628. doi: 10.1371/journal.pone.0308628 (PMC11530055; doi:10.1371/journal.pone.0308628)
Supplement: S4 Table — (PDF) [file pone.0308628.s004.pdf]

S4 Table. Estimators of Within-Study Variance

(1) $s^2_{C.j} = \tilde{n_j} + \frac{\hat{\delta}^2_{C.j}}{2(n_{1j}+n_{2j})}$	(13) $s^2_{C.j} = \tilde{n_j} + \frac{\gamma_j \hat{\delta}^2_{C.j}}{(n_{1j}+n_{2j}-2)}$
(2) $s^2_{H.j} = J_j^2 \tilde{n_j} + \frac{\hat{\delta}^2_{H.j}}{2(n_{1j}+n_{2j})}$	(14) $s^2_{H.j} = J_j^2 \tilde{n_j} + \frac{\gamma_j \hat{\delta}^2_{H.j}}{(n_{1j}+n_{2j}-2)}$
(3) $s^2_{H.j} = \tilde{n_j} + \frac{\hat{\delta}^2_{H.j}}{2(n_{1j}+n_{2j})}$	(15) $s^2_{H.j} = \tilde{n_j} + \frac{\gamma_j \hat{\delta}^2_{H.j}}{(n_{1j}+n_{2j}-2)}$
(4) $s^2_{C.j} = \frac{(n_{1j}+n_{2j}-2)}{(n_{1j}+n_{2j}-4)} \left(\tilde{n_j} + \frac{\hat{\delta}^2_{C.j}}{2(n_{1j}+n_{2j})} \right)$	(16) $s^2_{C.j} = \frac{(n_{1j}+n_{2j}-2)}{(n_{1j}+n_{2j}-4)} \left(\tilde{n_j} + \frac{\gamma_j \hat{\delta}^2_{C.j}}{(n_{1j}+n_{2j}-2)} \right)$
(5) $s^2_{H.j} = \frac{(n_{1j}+n_{2j}-2)}{(n_{1j}+n_{2j}-4)} \left(J_j^2 \tilde{n_j} + \frac{\hat{\delta}^2_{H.j}}{2(n_{1j}+n_{2j})} \right)$	(17) $s^2_{H.j} = \frac{(n_{1j}+n_{2j}-2)}{(n_{1j}+n_{2j}-4)} \left(J_j^2 \tilde{n_j} + \frac{\gamma_j \hat{\delta}^2_{H.j}}{(n_{1j}+n_{2j}-2)} \right)$
(6) $s^2_{H.j} = \frac{(n_{1j}+n_{2j}-2)}{(n_{1j}+n_{2j}-4)} \left(\tilde{n_j} + \frac{\hat{\delta}^2_{H.j}}{2(n_{1j}+n_{2j})} \right)$	(18) $s^2_{H.j} = \frac{(n_{1j}+n_{2j}-2)}{(n_{1j}+n_{2j}-4)} \left(\tilde{n_j} + \frac{\gamma_j \hat{\delta}^2_{H.j}}{(n_{1j}+n_{2j}-2)} \right)$
(7) $s^2_{C.j} = \tilde{n_j} + \frac{\hat{\Delta}^2_C}{2(n_{1j}+n_{2j})}$	(19) $s^2_{C.j} = \tilde{n_j} + \frac{\gamma_j \hat{\Delta}^2_C}{(n_{1j}+n_{2j}-2)}$
(8) $s^2_{H.j} = J_j^2 \tilde{n_j} + \frac{\hat{\Delta}^2_H}{2(n_{1j}+n_{2j})}$	(20) $s^2_{H.j} = J_j^2 \tilde{n_j} + \frac{\gamma_j \hat{\Delta}^2_H}{(n_{1j}+n_{2j}-2)}$
(9) $s^2_{H.j} = \tilde{n_j} + \frac{\hat{\Delta}^2_H}{2(n_{1j}+n_{2j})}$	(21) $s^2_{H.j} = \tilde{n_j} + \frac{\gamma_j \hat{\Delta}^2_H}{(n_{1j}+n_{2j}-2)}$
(10) $s^2_{C.j} = \frac{(n_{1j}+n_{2j}-2)}{(n_{1j}+n_{2j}-4)} \left(\tilde{n_j} + \frac{\hat{\Delta}^2_C}{2(n_{1j}+n_{2j})} \right)$	(22) $s^2_{C.j} = \frac{(n_{1j}+n_{2j}-2)}{(n_{1j}+n_{2j}-4)} \left(\tilde{n_j} + \frac{\gamma_j \hat{\Delta}^2_C}{(n_{1j}+n_{2j}-2)} \right)$
(11) $s^2_{H.j} = \frac{(n_{1j}+n_{2j}-2)}{(n_{1j}+n_{2j}-4)} \left(J_j^2 \tilde{n_j} + \frac{\hat{\Delta}^2_H}{2(n_{1j}+n_{2j})} \right)$	(23) $s^2_{H.j} = \frac{(n_{1j}+n_{2j}-2)}{(n_{1j}+n_{2j}-4)} \left(J_j^2 \tilde{n_j} + \frac{\gamma_j \hat{\Delta}^2_H}{(n_{1j}+n_{2j}-2)} \right)$
(12) $s^2_{H.j} = \frac{(n_{1j}+n_{2j}-2)}{(n_{1j}+n_{2j}-4)} \left(\tilde{n_j} + \frac{\hat{\Delta}^2_H}{2(n_{1j}+n_{2j})} \right)$	(24) $s^2_{H.j} = \frac{(n_{1j}+n_{2j}-2)}{(n_{1j}+n_{2j}-4)} \left(\tilde{n_j} + \frac{\gamma_j \hat{\Delta}_H^{\,2}}{(n_{1j}+n_{2j}-2)} \right)$

where $\hat{\delta}_{C.j}$ denotes by Cohen's d estimator, and $\hat{\delta}_{H.j}$ denotes by Hedges' g estimator. The $\hat{\Delta}_C$ denotes the overall effect size by Cohen's d estimator, and $\hat{\Delta}_H$ denotes the overall effect size by Hedges' g estimator. $\tilde{n_j} = 1/n_{1j} + 1/n_{2j}$, and $\gamma_j = (n_{1j} + n_{2j} - 2) - (n_{1j} + n_{2j} - 4)/J_j^2$.
